# Epidemiological, haematological-biochemical, and molecular investigations of bovine theileriosis with therapeutic evaluation of buparvaquone with silymarin in Dakahlia and Damietta governorates, Egypt

**DOI:** 10.1007/s11259-026-11089-4

**Published:** 2026-03-13

**Authors:** Mohamed Gamal Alnahass, Ahmed Magdy Selim, Mohamed El-Diasty, Ahmed Mohamed El-Sebaey, Elzahara Elbaz

**Affiliations:** 1 Department of Internal Medicine and Infectious Diseases, Faculty of Veterinary Medicine, Mansoura University, Mansoura, 35516 Egypt; 2 Agricultural Research Center (ARC), Animal Health Research Institute-Mansoura Provincial Laboratory (AHRI-Mansoura), Giza, 12618 Cairo Egypt; 3 Department of Clinical Pathology, Faculty of Veterinary Medicine, Mansoura University, Mansoura, Egypt

**Keywords:** *Theileria annulata*, Epidemiology, PCR, Phylogenetic analysis, Buparvaquone, Silymarin

## Abstract

**History:**

Bovine theileriosis, caused by *Theileria annulata* and transmitted by Hyalomma ticks, is a major constraint on cattle health and productivity in Egypt, leading to economic losses.

**Aims:**

This study determines the molecular prevalence of *T. annulata* in cattle from Dakahlia and Damietta, evaluates associated risk factors, and assesses hematological and biochemical changes in infected animals. The therapeutic efficacy of buparvaquone (BVQ) alone or combined with silymarin (SI) was also investigated.

**Methods:**

Blood samples were collected from 149 cattle; infection was confirmed by *Tams1* gene PCR and phylogenetic analysis. Risk factors were statistically assessed. Twenty clinically infected cows were divided into two groups (*n* = 10); one received BVQ, while the other received BVQ plus SI. Hematological, biochemical, acute-phase proteins, and beta-hydroxybutyrate were measured before and two weeks after treatment.

**Results:**

PCR analysis confirmed *Theileria annulata* infection in 32.9% of cattle, with our isolates (PQ137836 and PQ137837) showing 99.7–100% sequence similarity to both local and global strains. Infection was highest in tick-infested cattle (69.84%, OR = 5.62), pregnant cows (50%, OR = 6.7), and animals not treated with ectoparasiticides (91.8%, OR = 8.6). Combination therapy significantly reduced parasitemia (3.76% to 0.18%, *p* < 0.001), improved hematological, liver, and metabolic indices, decreased APPs, and increased paraoxonase-1 activity, compared to BVQ alone.

In conclusion.

Bovine theileriosis is highly prevalent in Dakahlia and Damietta, particularly in middle-aged, pregnant, and tick-infested cattle during the summer. Molecular analysis confirmed *Theileria annulate* infection. Combination therapy with BVQ and SI proved more effective than BVQ monotherapy.

## Introduction

Bovine theileriosis, caused by the protozoan parasite *Theileria annulata*, is a prominent tick-borne disease of cattle that has serious economic and health consequences in Egypt and other subtropical and tropical areas (Al-Hosary et al. 2024; Khalifa et al. 2025). The disease is largely transmitted by ticks of the Hyalomma genus, which are found throughout North Africa, the Middle East, and Asia (El-Dakhly et al. 2018). Theileriosis is a constant hazard to cattle productivity in Egypt, affecting both milk and meat production, and is particularly significant in governorates located in the Nile Delta region because of the suitable ecological circumstances for tick proliferation (Al-Hosary et al. 2024). Approximately 250 million cattle globally are at risk from *T. annulata*, and yearly global losses owing to ticks and tick-related diseases in these cattle are estimated to be between US$13.9 and US$18.7 billion (Kasozi et al. 2014; Uztimür et al. 2025). Thousands of cattle in Egypt lose their lives to *T. annulata* every year, which results in significant output losses and high costs for veterinary care and tick control. The economic impact of tick infestation and *T. annulata* is estimated to range from 3.1 to 57.2 million US dollars annually in Egypt (Al-Hosary et al. 2024).

An advantageous understanding of the epidemiology and risk factors of *T. annulata* infection is crucial for the effective control and management of tropical theileriosis in cattle (Madkour et al. 2023)**.** In fact, incorporating epidemiological and risk factor statistical results into disease control strategies results in more long-term reductions in morbidity, mortality, and economic losses caused by bovine theileriosis (Kumar et al. 2018)**.** Clinical signs begin with high fever, lymphadenitis, systemic signs, decreased milk production, constipation, followed by dark tarry diarrhea in severe cases, along with anemia (Madkour et al. 2023; Ghani et al. 2025)**.**

Parasitic detection in peripheral blood smear is the most commonly used diagnostic approach. Despite this, this method is inapplicable in situations with low infection rates and coinfection, when it is challenging to distinguish between *Theileria* species spp (Clift et al. 2020)**.** Polymerase Chain Reaction (PCR) is the confirmative diagnostic method for *Theileria* spp. for its high sensitivity and specificity (Hassan et al. 2024)**.** In outbreaks, combining PCR with phylogenetic analysis of different genes allows for a more comprehensive understanding of pathogen genetic variation (El-Alfy et al. 2022; Mans 2022)**.**

Theileriosis in cattle is treated with a number of allopathic drugs, including parvaquone, buparvaquone (BVQ), halofuginone lactate, oxytetracycline, and diminazene aceturate (Prajapati et al. 2022). The best therapeutic strategy for controlling theileriosis is thought to be an intramuscular injection of BVQ at a single dose of 2.5 mg/kg body weight (BW) (Neamat-allah 2016; Silatsa et al. 2020). Liver involvement, which frequently appears as jaundice, hemolytic anemia, hypoproteinemia, negative energy balance, and ketosis, is a characteristic of *T. annulata*-induced bovine theileriosis (Abou-El-Naga et al. 2005).

The best outcome would be achieved by concurrently managing the liver condition while treating *Theileria*. Therefore, using hepatoprotectants is essential to manage liver disease. They may be herbal or natural, like silymarin (SI). The seeds of Silybum marianum L. (SI) Gaertn have been used to improve bile duct and liver health and cure a variety of liver and gallbladder conditions, such as cirrhosis, icterus, and hepatitis. More recently, there has been a noticeable increase in the liver's defense against environmental and chemical poisoning, such as that caused by any disease (Radko and Cybulski 2007).

Several investigations on animals with liver disorders have shown that SI therapy has favorable effects, including increased protein synthesis as well as anti-inflammatory, immunomodulatory, antifibrotic, and antioxidant properties (Ulger et al. 2017)**.** Silymarin also inhibits the absorption of poisons by hepatocytes (Khazaei et al. 2022) implying potential usefulness in the treatment of liver damage caused by medicines or toxic substances. This study aimed to determine the molecular prevalence of *T. annulata* infection in both symptomatic and asymptomatic cattle in Dakahlia and Damietta Governorates around the highly endemic Nile Delta region. Additionally, the objective is extended to evaluate the enhancement of BVQ therapeutic efficacy in controlling parasitemia, hematological and biochemical changes, and acute phase proteins (APPs) by incorporating SI into the therapeutic approach.

## Material and methods

### Animals

One hundred forty-nine cattle, ranging from less than 1 year to more than 8 years old, reared in private Egyptian farms in the Dakahlia and Damietta governorates, were sampled and inspected during the period from August 2023 to September 2024. A variety of epidemiological and clinical data, including age, breed, sex, season, tick infestation, contact with cattle from different herds during grazing, lactation and pregnancy status, history of clinical signs in the past, and the number of ectoparasiticide applications made annually, were collected using questionnaires and farm records.

### Treatment trial

A total of 49 animals ranging from 2.50 to 6 years were confirmed to be infected with *Theileria annulata* based on PCR and/or microscopic examination of blood smears. From this, 20 animals that fulfilled the eligibility criteria were randomly assigned to two groups (n = 10 each). The first group received buparvaquone (BVQ) administered intramuscularly as a single dose of 2.5 mg/kg body weight. The second group received BVQ (2.5 mg/kg, intramuscularly) combined with silymarin (SI, 2.5 mg/kg, intramuscularly) as supportive therapy to enhance hepatic function and recovery, following Tedesco et al. (2004). Both groups were used to evaluate baseline parasitemia%, hematological, and biochemical parameters at 0- and 14-day post-treatment trials. Our laboratory's standardized reference intervals have also been added to assess the efficacy of treatment regimen values and to identify results that fall within or outside the reference interval (ORI).

Animals were included if they had not received any treatment since clinical diagnosis, were infected exclusively with *T. annulata*, and were clinically stable enough to complete the 14-day follow-up period without requiring additional therapeutic interventions. Animals were excluded if they lacked complete hematological or biochemical data, gave birth during the study period, received drugs related to the drying-off period or external tick control, or required additional medications beyond BVQ and/or SI to ensure survival according to ethical regulations.

### Sample collection and processing

Peripheral blood samples were obtained from the tail vein. After this, samples were placed in an insulated box with reused ice during shipping to the clinical pathology laboratory for processing and analysis within 12 h (Jain 1993). Anticoagulated whole blood samples were added to Tri Potassium-EDTA in 5 ml VACUETTE® tubes (Greiner Bio-One, GmbH, Germany) for molecular diagnosis of theileriosis, as well as immediate blood smear preparation to estimate parasitemia % estimation and manual blood count at zero day and at the end of the treatment trial (14 days). For serum separation, additional blood samples were collected in 10 ml plain tubes with clot activator (VACUETTE®, Greiner Bio-One, GmbH, Germany) and kept in the refrigerator (4 °C) for 15 min till complete clot retraction, and the serum sample was separated by centrifugation at 2800 × g for 10 min at 4 °C (REMI Refrigerated centrifuge, C-24BL, Maharastra, India) and labelled correctly in sterile plastic Eppendorf microtubes (Thrall et al. 2015) for prospective analysis of hepatobiliary, renal, metabolic, and ketogenesis proteins and APPs markers.

### Laboratory investigation

#### Erythrocyte infection intensity (Parasitaemia %)

Two slides per animal were smeared using tri-potassium-EDTA-whole-blood samples. After air-drying, the prepared smears were fixed in methanol for 2 min and then stained with Giemsa stain at a concentration of 10% for 10–15 min. Giemsa-stained blood smears were examined for the better visualization of erythrocyte infection rate with intracellular comma-shaped and pear-shaped piroplasms of *T. annulata*. For each smear, 50 high-power microscopic fields (HPF) were thoroughly examined and photographed under an oil immersion lens (100X) of the **OPTIKA** **B**-**380** series light microscopy (Bergamo, Italy), and parasitemia % was computed according to Elati et al. (2023) as follows:

$$\text{Parasitaemia }\left(\mathrm{\%}\right)=\frac{\text{Number of infected RBCs}}{\text{Total number of RBCs per }50\text{ HPF}}\times 100$$

If a red blood cell has several parasites, it is counted as one infected RBC.

#### Hematological profile

The total red blood cell (RBC) and leukocyte (WBC) counts were manually estimated using two chambered Neubauer-improved hemocytometers (Germany) (Sirois 2014). Diff-Quick-stained blood smears were prepared and microscopically visualized for the relative differential leukocytic count. To simplify data interpretation, the absolute differential WBC count was derived from using the relative one and the total WBC count based on the rule of three. Furthermore, spectrophotometric quantitative determination of haemoglobin (Hb) was performed using the colourimetric endpoint method with Drabkin’s Reagent (VITRO SCIENT, Hannover, Germany, REF# 1701), as outlined by the supplier. The relatively packed erythrocyte volume (PCV%) was estimated after performing the microhematocrit method. In addition, indices of erythrocyte size (MCV) and Hb concentration (MCH and MCHC) were calculated using RBC, Hb, and PCV% parameters, following standard equations provided by Sirois (2014) as follows:

$$\begin{array}{c}\text{MCV }(\mathrm{fL}) = (\text{PCV \% }/\text{ RBC count }\times {10}^{12}/\mathrm{L})\text{ x }10\\ \text{MCH }(\mathrm{pg}) = (\text{Hemoglobin g}/\text{dL }/\text{ RBC count }\times {10}^{12}/\mathrm{L}) \times 10\\ \text{MCHC }(\mathrm{\%}) = (\text{Hemoglobin g}/\text{dL }/\text{ PCV \%}) \times 100\end{array}$$

#### Serum metabolic status parameters

Metabolic parameters including alanine aminotransferase (ALT, Code # 51409002), aspartate aminotransferase (AST, Code # 51408002), γ-glutamyl transferase (Ɣ-GT, Code # 51416002), alkaline phosphatase (ALKP, Code # 51401002), and total bilirubin (T. Bilirubin, Code # 51003002) as Liver integrity and clearance function indicators, as well as Urea (Code # 51412002), and creatinine (Code # 51009001) as representative hepatic and renal function tests, in addition to glucose (Code # 51406001), Cholesterol (Code # 51204002), and Triglycerides (Code # 51410005) as biomarkers of negative energy balance and ketogenesis, were estimated by using commercial kits of the same brand (AGAPPE ®, Ernakulam, Kerala, India) with the aid of a semi-automatic chem-7 spectrophotometer (Erba Mannheim, Germany) as outlined by the supplier company.

For estimation of β-hydroxybutyrate (BHB), which accounts for about 75% of the total ketone body, commercial colorimetric reagents exhibiting optimal inter- and intra-assay precision (CV < 4%) were purchased from Elabscience® (Houston, USA) and subsequently revalidated for the assessment of BHB (Cat # E-BC-K785-M) by quantifying the variation in absorbance at 450 nm in line with the manufacturer's instructions.

#### Proteinogram

The concentration of positive acute phase α-glycoproteins, such as α1-acid glycoprotein (α1-AGP) and α2-glycoprotein (haptoglobin, HP), was estimated by Hoelzel Diagnostika® using bovine-specific α1-AGP (Cologne, Germany, Cat # CSB-E13213G) and HP (Cologne, Germany, Cat # CSB-E08579b) ELISA kits. All tested samples, standards, and controls were assayed in duplicate at 450 nm. For α1-AGP, the detection range was 62.5–4000 ng/mL, the lower detection limit was 15.6 ng/mL, and the intra- and inter-assay precision (CVs%) were < 8 and < 10%, respectively. For HP, the detection range was 7.81–2000 ng/mL, the lower detection limit was 7.81 ng/mL, and the intra- and inter-assay precision (CVs%) were < 8 and < 10%, respectively.

The serum levels of total protein (T. Protein, Code # 51,013,002) and albumin (Code # 51,001,002), as negative acute phase reactants, were estimated by colourimetric methods using AGAPPE diagnostic kits. In addition, the serum activity of HDL-C-attached paraoxonase-1 (PON-1) as negative APPs was estimated as U/L, depending on measuring the rate of para-nitrophenol end-product formation at 405 nm following Eckerson et al. (1983) and Razgildina et al. (2018) previously validated protocol. Finally, the globulin was computed by subtracting albumin from the total protein to obtain the A/G ratio value.

### Molecular analysis

#### DNA extraction and amplification

Genomic DNA was extracted from 200 µl of whole blood from individual cattle using the commercially available QIAamp DNA Mini Kit according to the manufacturer's instructions. The quality and integrity of the DNA were verified using UV/Visible NanoDrop 2000 spectroscopy (Thermo Fisher Scientific) and by electrophoresing the genomic DNA on a 0.8% agarose gel. After the quality check, DNA samples were stored at − 80 °C until further experiments. *T. annulata*-specific *Tams1* gene primers were used for conventional PCR with a set of primers (*Tams1*-F GTAACCTTTAAAAACGT and *Tams1*-R GTTACGAACATGGGTTT) that amplified a 721-bp fragment (Nourollahi-Fard et al. 2015).

The PCR reactions were conducted in a total volume of 25 ml, containing 12.5 μl of Emerald Amp GT PCR mastermix (2 × premix), 1 μL of forward primer (20 pmol)**,** 1 μLof reverse primer (20 pmol)**,** 5 μl of Template DNA, and 5.5 μL of PCR-grade water.

Amplifications were performed using the Thermal Cycler (T100TM Thermal Cycler, Bio-Rad, USA) under the conditions started with one-step initial denaturation at 94˚C for 5 min, followed by 35 cycles of denaturation at 94˚Cfor 30 s, annealing at 55˚C for 40 s, and extension at 72˚C for 45 s, with a final extension at 72˚C for 10 min for *T.annulata*.

The PCR products were subjected to horizontal electrophoresis on a 1.5% agarose gel containing ethidium bromide dye (Himedia, India) in Tris–acetate-EDTA (TAE) buffer at 120 V for 30 min and visualised under a UV light gel documentation system (Gel DocTM EZ Imager, Bio-Rad, Hercules, CA, USA). From positive samples of *T.annulata*, a highly bright and thick band was selected for DNA sequencing.

#### DNA nucleotide sequencing

The purification of PCR products was carried out through the QIAquick PCR Product extraction kit*.* (Qiagen Inc., Valencia, CA, Cat # 28,104), and sequencing reactions occurred through using the BigDye Terminator V3.1 cycle sequencing kit*.* (Perkin-Elmer, Foster City, CA, Cat # 4,336,817). Sequence analysis was done using an Applied Biosystems 3130 genetic analyzer (HITACHI, Japan). The sequence was then purified by a Centrisep spin column (Cat # CS-901).

#### Phylogenetic analysis

The nucleotide sequence was compared with previously available Tams 1 gene sequences of *T. annulata* parasites in the National Center for Biotechnology Information (NCBI) database using the basic local alignment search tool (BLAST) followed by multiple alignments using ClustalW2. Our isolated nucleotide sequence was deposited in the NCBI GenBank database with the accession numbers (PQ137836 and PQ137837). The phylogenetic tree was constructed based on a maximum likelihood phylogeny, Tamura 3 parameter model and 1000 bootstrap replicate using MEGA 7 (Tamura et al. 2013).

#### Statistical analysis and data illustration

Raw data were analysed using SPSS V20.0 software (IBM Corp., NY, USA). Data homoscedasticity (Gaussian distribution) was confirmed by the Shapiro–Wilk test, which showed that the probability value (*P*) was greater than 0.05, confirming the null hypothesis that the data are normally distributed. Erythrocyte infection rate (parasitemia %) and estimated hematobiochemical parameters in treatment groups were represented as mean ± standard deviation (SD). The significant difference of the dataset before (Day 0) and after treatment (Day 14) for the same treatment group was evaluated using a Paired Sample T-test, and a value of *P* < 0.05 was considered statistically significant as per the principles of (Snedecor and Cochran 1994). Illustrated graphs of parasitemia % and serum biochemical parameters were generated by GraphPad Prism 9.5.1 (CA, USA) and OriginPro (Origin Lab 2022) software, respectively. Logistic regression analysis was performed to check the relationship between the prevalence of infection and the potential risk factors. The univariable binary logistic regression statistics followed by the independent factors with significant *P*-value (*P* < 0.05) were incorporated into the multivariable binary logistic regression analysis. For each variable, the results were P value, confidence interval (CI: 95%), and odds ratio (OR). Results were considered to be significant at *P* < 0.05 (Rizk et al. 2017).

## Results

### Epidemiological results

Epidemiological information in Table 1 shows data acquired on the animals. The study included cattle aged from less than one year to 8 years old. Young cows (1–3 years) had the highest prevalence (61.9%), followed by adult cows (3–5 years) (34.6%) and the lowest prevalence in cows with age 5–8 years (12.1%). The univariable binary logistic regression model revealed statistically significant results for all variables except location, species, and sex (*p* > 0.05). On the other hand, there was a statistically significant association between the number of ectoparasiticide treatments received annually and the prevalence of theileriosis (*p* < 0.05). The group that did not use ectoparasiticides had the highest infection rate (68.2%, *p* ≤ 0.001, OR = 2.25, 95% CI: 007-0.070.070). Pregnancy was found to be statistically associated with the prevalence of theileriosis; pregnant cows (50%), (*p* ≤ 0.001, OR = 4.4), and the prevalence was higher in clinically affected animals with LN enlargement (73.8%, *p* ≤ 0.001, OR: 62.25, 95% CI: 19.493–198.788) than in asymptomatic ones. There was also a statistically significant association between the occurrence of *Theileria* and tick presence; the highest prevalence was observed in animals with tick infestation (69.84%, *p* ≤ 0.001, OR = 37.5, 95% CI: 13.111–107.345) additionally, there was also a statistically significant association between the occurrence of *Theileria* and eye lacrimation, the highest prevalence was observed in animals with eye lacrimation (65.1%, *p* ≤ 0.001, OR: 0.103, 95% CI: 7.756–46.718). Fever was important symptom associated with theileriosis, out of 61 animals showing fever, 45 (73.8%) were confirmed positive for *Theileria* by PCR (73.8%, *p* ≤ 0.001, OR: 63,95%CI: 19.733–201.136) and 4(4.55%) not infected.

**Table 1:** Results of binary logistic regression analysis to test the association between the occurrence of theileriosis and associated risk factors

Variable	Categories	Number of animals (%)	Number of positive cases by PCR (%)	OR	*P-*Value	CI
Age					0.001***	
	1 year	37 (24.8)	4 (10.8)	2.061	0.532	0.213–19.907
	1–3 years	42 (28.2)	26 (61.9)	27.625	0.002**	3.347–228.029
	3–5 years	52 (34.9)	18 (34.6)	9	0.04*	1.106–73.213
	5–8 years	18 (12.1)	1 (5.56)	-	-	-
Species	Cattle	55 (36.9)	22 (40)	1.654	0.159	0.821–3.333
	Buffalo	94 (63.1)	27 (28.7)	0.403	-	-
Sex	Male	84 (56.4)	26 (40)	1.768	0.106	0.887–3.525
	Female	65 (43.6)	23 (27.4)	-	-	-
Season	Summer	93 (62.4)	41 (44.1)	4.731	0.001***	2.016–11.103
	Winter	56 (37.6)	8 (14.3)	-	-	-
Eye Cataract	Yes No	65 (43.6)	45 (69.23)	47.368	0.001***	15.174–147.872
		84 (56.4)	4 (4.76)	-	-	-
Fever	Yes No	61 (40.9)	45 (73.8)	63	0.001***	19.733–201.136
		88 (59.1)	4 (4.55)	-	-	-
Lymph nodes Enlargement	Yes No	61 (40.9)	45 (73.8)	62.25	0.001***	19.493–198.788
		88 (59.1)		-	-	-
Pregnancy	Pregnant Not pregnant	68 (45.6)	34 (50)	4.4	0.001***	2.11–9.176
		81 (54.4)	15 (18.52)	-	-	-
Off food	Yes	80 (53.7)	40 (50)	6.838	0.001***	2.988–15.648
	No	69 (46.3)	9 (13)	-	-	-
Location	El Dakahlia	90 (60.4)	30 (33.3)	1.053	0.886	0.523–2.12
	Damietta	59 (39.6)	19 (32.2)	-	-	-
Eye lacrimation	Yes	63 (42.3)	41 (65.1)	19.036	0.001***	7.756–46.718
	No	86 (57.7)	8 (9.3)	-	-	-
Presence of ticks	Yes	63 (42.3)	44 (69.84)	37.516	0.001***	13.111–107.345
	No	86 (57.7)	5 (5.81)	-	-	-
Reduction of milk production	Reduction	69 (46.3)	35 (50.7)	4.853	0.001***	2.303–10.225
	Normal	80 (53.7)	14 (17.5)	-	-	-
Ectoparasitic treatment	Without	66 (44.3)	45 (68.2)	2.250	0.003*	0.007–0.070
	Yes	83 (55.7)	4 (4.82)	-	-	-

*S.E* standard error *P < 0.005; *CI* confidence interval, *OR* odds ratio, *G* group; the superscript (*) indicates significant change; ^*^P ≤ 0.05, ^**^P ≤ 0.01, and ^****^P ≤ 0.001

The multivariable binary logistic regression model (Table 2) showed that season, eye cataract, pregnancy, presence of ticks and frequency of ectoparasiticide application per year were the potential risk factors for the occurrence of tropical theileriosis. The season revealed a significant association with a high prevalence of the infection (*P* = 0.03; OR: 6.97; 95% CI: 1.1- 43.9), furthermore, pregnancy revealed a significant association with a high prevalence of the infection (*P* = 0.009; OR: 6. 7; 95% CI: 1.6- 27.9). Additionally, the higher prevalence was observed in the group that used ectoparasiticides (*P* = 0.06; OR: 8.6; 95% CI: 1.86–39.63).

**Table 2:** Results of multivariable binary logistic regression analysis to test the association between the occurrence of theileriosis and associated risk factors

Variable	Categories	OR	*P-*Value	CI
Season	Summer	6.97	0.03*	1.10 - 43.94
	Winter	-	-	-
Eye Cataract	Yes	6.3	0.01**	1.41 - 28.29
	No	-	-	-
Pregnancy	Pregnant	6.7	0.009*	1.60 - 27.9
	Not pregnant	-	-	-
Presence of ticks	Yes	5.62	0.04*	1.08 - 29.23
	No	-	-	-
Ectoparasitic treatment	Without	8.6	0.006**	1.86 - 39.63
	Yes	-	-	

*P < 0.005; *CI* confidence interval, *OR* odds ratio, *G* group; the superscript (*) indicates significant change; ^*^P ≤ 0.05, ^**^P ≤ 0.01, and ^****^P ≤ 0.001

### Hematological and biochemical responses to BVQ treatment and BVQ + SI combination therapy

**Fig. 1:**
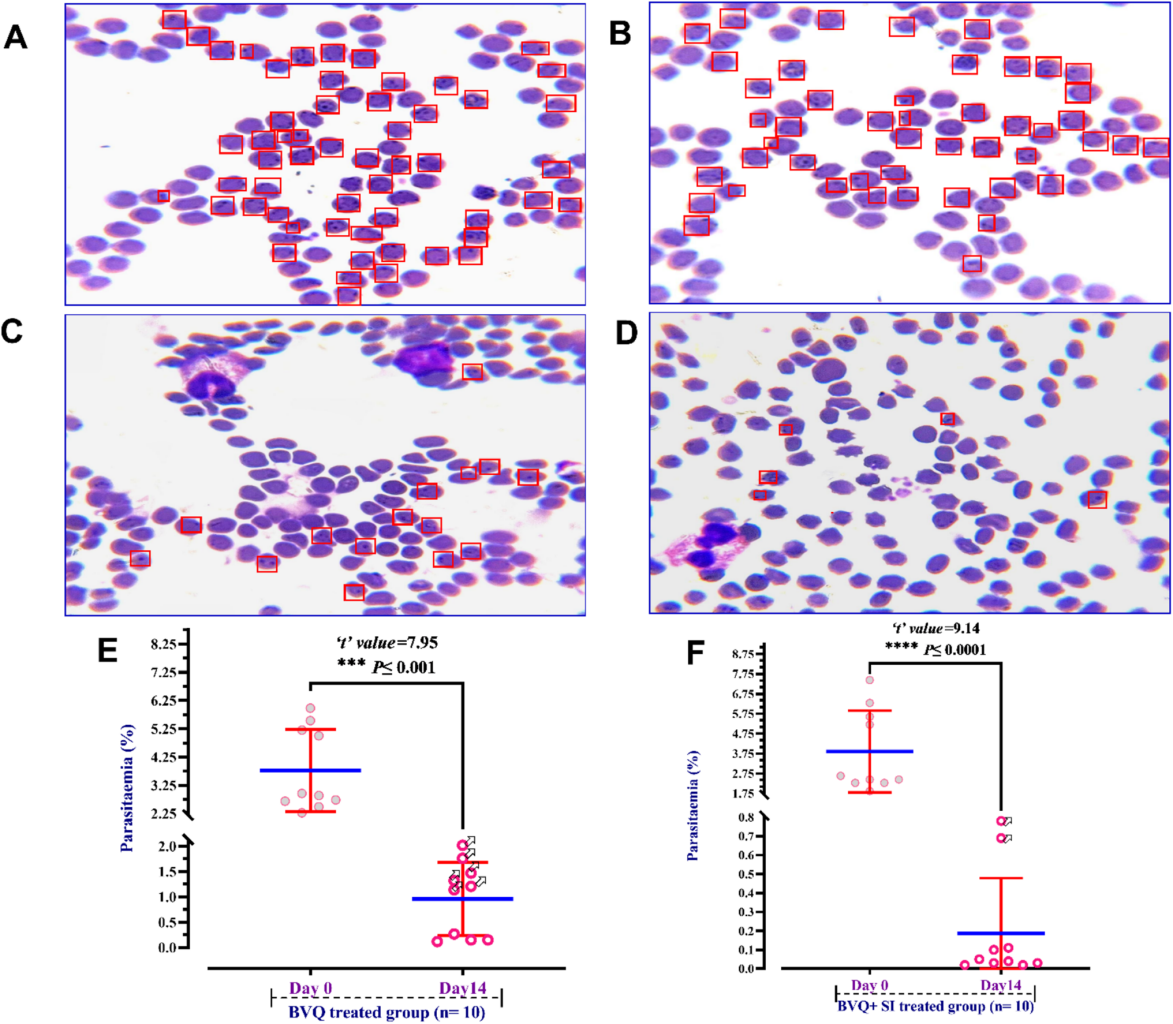
Giemsa stained blood smears (**A**-**D**) depict the 2–7 polymorphic, comma-shaped, and pear-shaped piroplasm-like forms of *T. annulata* parasite (red rectangle) within the infected bovine RBCs (1000X) before (**A** & **B**) and after a two-week treatment with 2.5 mg/kg BVQ (**C**) or a combination of 2.5 mg/kg BVQ and 2.23 mg/kg SI (**D**). The interleaved scatter plot illustrates the variations in parasitemia% before and after treatment within the same studied group (**E** & **F**). Circles denote the parasitaemia % for each case in each group. Red error bars represent ± standard deviation of the mean, whereas the blue line indicates the mean parasitemia % for each group. Paired Student’s *t*-test was used for comparisons of means in pretreatment day 0 and posttreatment day 14 within the same treatment group, with the significance levels versus pretreatment day 0 are as follows: *** *P* ≤ 0.001; **** *P* ≤ 0.0001

The pre-treatment (Day 0) stained blood smears in Fig. 1 show an elevated bovine erythrocyte infection rate (parasitemia %) with 2–7 small (0.5–2 µm), polymorphic, comma-shaped, pear-shaped *T. annulata* parasites in the BVQ (Fig. 1A) or BVQ + SI (Fig. 1B) groups. The mean parasitemia percentages were 3.78 ± 1.46 and 3.76 ± 1.35% in the studied groups, indicating no significant differences between the two groups at baseline. Following two weeks of treatment, a significant decrease in parasitemia % was noted in the blood smears examined from both groups (Fig. 1C and D). In the BVQ-treated group (Fig. 1E), the erythrocyte infection rate decreased to a mean of 0.96 ± 0.72%, as evidenced by the paired t-test. Notwithstanding this reduction, six cows in this cohort demonstrated persistent high parasitemia (denoted by six white arrows), indicating partial resistance to BVQ monotherapy. Indeed, the combination (BVQ + SI) group achieved a more significant reduction in parasitemia% and reached a mean of 0.18 ± 0.29 at Day 14 of treatment (Fig. 1F). Only two high parasitemia cases persisted (marked by two white arrows), emphasizing the superior efficacy of the combination therapy in managing *T. Annulata* infections.

Table 3 demonstrates the enhanced efficacy of combination therapy, BVQ + SI, over BVQ monotherapy in achieving superior hematological recovery in naturally infected cattle with *T. annulata*. Despite a modest yet statistically significant enhancement (*P* < 0.05) in hematological parameters following 14 days of treatment with 2.5 mg/kg BVQ, the mean values of RBCs, Hb, PCV, WBCs, and lymphocytes remained outside the reference interval (ORI). The persistence of ORI values and BVQ-resistant cases in naturally infected cattle with *T. annulata* suggests that BVQ monotherapy is insufficient to fully correct hematological abnormalities (normocytic normochromic anemia, leukopenia, and lymphopenia) obtained before treatment (Day 0). Notably, pretreatment (Day 0) hematological abnormalities, specifically normocytic normochromic anemia and leukopenia characterized by lymphopenia and neutropenia, were substantially mitigated after two weeks of treatment with a combination of 2.5 mg/kg BVQ and 2.23 mg/kg SI, as demonstrated by significant enhancements in their levels (*P* = 0.005). Additionally, pseudo macrocytosis (reticulocytosis) as a regenerative bone marrow response sign was confirmed by the significant (*P* < 0.05) increase in MCV (macrocytosis) and decrease in MCHC (hypochromasia), indicating the release of macrocytic hypochromic reticulocyte cells in peripheral blood. Thus, the ORI count for most hematological parameters markedly improved, with only two cases exhibiting resistance.

**Table 3:** Haematological variables of naturally infected cattle with *T. Annulata* before and after a two-week treatment with 2.5 mg/kg BVQ or a combination of 2.5 mg/kg BVQ and 2.23 mg/kg SI

Parameters	Reference Interval (RI)	BVQ treated group (*n* = 10)					BVQ + SI treated group (*n* = 10)				
		Day 0		Day 14		*‘t*’ value *(P*-value)	Day 0		Day 14		*‘t’* value (*P*-value)
		ORI	Mean ± SD	ORI	Mean ± SD		ORI	Mean ± SD	ORI	Mean ± SD	
RBC (10^12^/L)	5–10	10	3.43 ± 0.44(**‣**)	6	4.97 ± 0.94(**‣**)	t = −2.93 *P* = 0.042^*^	10	3.73 ± 0.61(**‣**)	2	6.56 ± 1.47	t = −5.57 *P* = 0.005^**^
Hb (g/L)	8–15	10	5.82 ± 0.88(**‣**)	6	7.96 ± 1.52(**‣**)	t = −2.82 *P* = 0.048^*^	10	6.18 ± 1.13(**‣**)	2	10.59 ± 2.22	t = −5.51 *P* = 0.005^**^
PCV (%)	24–46	10	17.89 ± 1.86(**‣**)	6	23.46 ± 5.60(**‣**)	t = −2.96 *P* = 0.037^*^	10	16.77 ± 2.51(**‣**)	2	39.82 ± 9.38	t = −5.73 *P* = 0.005^**^
MCV (fl)	40–60	0	50.75 ± 4.11	0	49.11 ± 2.76	t = 1.11 *P* = 0.33^NS^	0	45.08 ± 2.79	2	61.26 ± 4.52(**‣**)	t = −3.65 *P* = 0.046^*^
MCH (pg)	11–17	0	16.90 ± 1.19	0	16.03 ± 0.18	t = 1.60 *P* = 0.19^NS^	0	16.59 ± 1.11	0	16.18 ± 0.48	t = 0.63 *P* = 0.56^NS^
MCHC (%)	30–36	0	33.37 ± 2.15	0	32.81 ± 2.03	t = 0.45 *P* = 0.68^NS^	2	36.01 ± 1.67	2	27.18 ± 4.53(**‣**)	t = 7.27 *P* = 0.002^**^
WBC (10^9^/L)	4–12	10	2.75 ± 0.51(**‣**)	6	3.98 ± 0.89(**‣**)	t = −3.22 *P* = 0.032^*^	10	2.77 ± 0.69(**‣**)	2	5.74 ± 0.92	t = −8.02 *P* = 0.001^***^
Neutrophil (10^9^/L)	0.6–4	3	0.56 ± 0.25(**‣**)	0	1.13 ± 0.33	t = −3.71 *P* = 0.021^*^	4	0.53 ± 0.16(**‣**)	0	1.60 ± 0.19	t = −5.73 *P* = 0.005^**^
Lymphocyte (10^9^/L)	2.5–7.5	10	1.71 ± 0.33(**‣**)	6	2.47 ± 0.58(**‣**)	t = −2.82 *P* = 0.048^*^	10	1.73 ± 0.34(**‣**)	2	3.81 ± 0.98	t = −5.59 *P* = 0.005*^*^
Monocyte (10^9^/L)	0.0–0.85	0	0.33 ± 0.061	0	0.27 ± 0.047	t = 2.14 *P* = 0.099^NS^	0	0.34 ± 0.051	0	0.26 ± 0.043	t = 13.38 *P* = 0.001^***^
Eosinophil (10^9^/L)	0.0–2.4	0	0.16 ± 0.051	0	0.11 ± 0.024	t = 3.44 *P* = 0.026^*^	0	0.17 ± 0.026	0	0.10 ± 0.013	t = 8.12 *P* = 0.001^***^

Paired Student’s *t*-test was used for comparisons of means in pretreatment day 0 and posttreatment day 14 within the same treatment groups. Significance versus pretreatment day 0: * *p* < 0.05; ** *p* ≤ 0.01; *** *p* ≤ 0.001. Values out of the reference interval are marked with the (**‣**) symbol. *ORI* number of cows that had values out of the reference interval, *RBC* red blood cells, *PCV* packed cell volume, *Hb* hemoglobin, *MCV* mean erythrocyte volume, *MCH* mean erythrocyte hemoglobin, *MCHC* mean erythrocyte hemoglobin concentration, and *WBC* white blood cell count

**Fig. 2:**
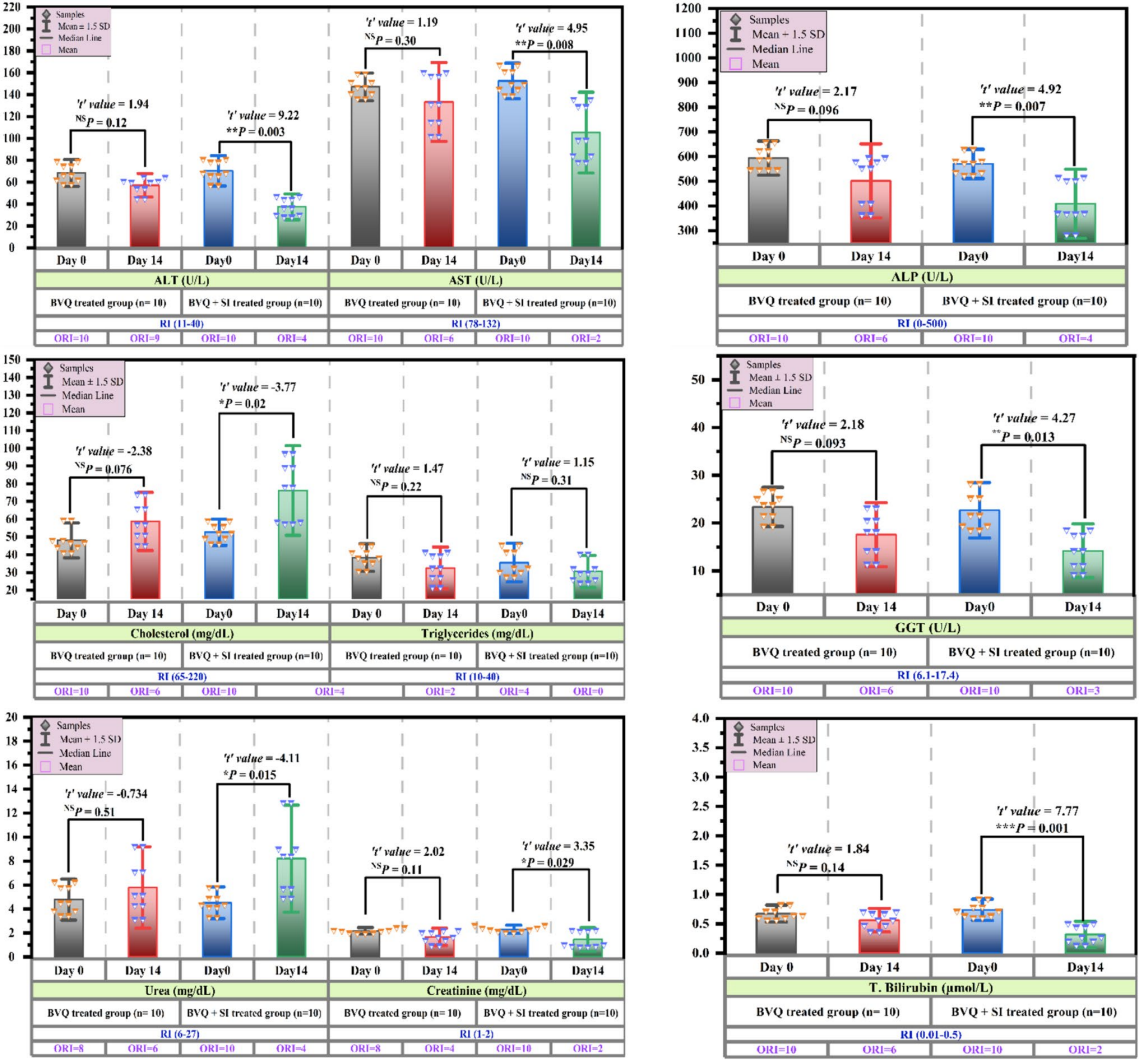
Bar plots depict the Mean ± SD of serum hepatobiliary, and renal biomarkers in cattle naturally infected with *T. annulata* before and after a two-week treatment with 2.5 mg/kg BVQ or a combination of 2.5 mg/kg BVQ and 2.23 mg/kgSI. Diamonds denote the individual value for each case in each group. Error bars represent ± standard deviation of the Mean, whereas the bar length indicates the mean value of each biomarker for each group. Paired Student’s *t*-test was used for comparisons of means in pretreatment Day 0 and posttreatment Day 14 within the same treatment group, with the significance levels versus pretreatment Day 0 as follows: ^NS^ *P* > 0.05; * *P* < 0.05; ** *P* ≤ 0.01; *** *P* ≤ 0.001. ALT: alanine aminotransferase; AST: aspartate aminotransferase; ALKP: alkaline phosphatase; GGT: Gamma-glutamyl transferase; T. bilirubin

The serum hepato-renal biochemical profile indicated in Fig. 2 reveals that hepatic (ALT, AST, T. Bilirubin) and biliary (ALP, GGT) biomarkers in cows infected with *T. annulata* remained elevated at Day 14 post-treatment with 2.5 mg/kg BVQ and showed no significant improvement (*P* > 0.05) from pretreatment levels on Day 0, with six cows surpassing the reference interval (RI). In stark contrast, the combination therapy of 2.5 mg/kg BVQ and 2.23 mg/kg SI for two weeks demonstrated enhanced efficacy in hepatic and biliary health compromised by *T. annulata* infection, as indicated by the significant (*P* ≤ 0.001) reduction in these parameters, normalizing them to within the RI for most cases. Nevertheless, three to four clinical cases continued to demonstrate values above the RI.

The outlined urea and creatinine levels in Fig. 2 reveal the impact of BVQ-based therapy on renal functions in *T. annulata*-infected cows. The low serum urea and high creatinine levels at Day 0 showed no statistically significant change (*P* > 0.05) post-treatment in the 2.5 mg/kg BVQ-treated group. The post-treatment group continued to display 6–4 cases with ORI values. On the other hand, creatinine levels were significantly (*P* = 0.029) lowered while urea levels significantly increased (*P* = 0.015) on Day 14 of treatment with the BVQ + SI combination. Furthermore, most cases in the combination group had both biomarkers normalized to fall within the RI, which shows that the combination therapy was more effective. There was some response variability, though, since 2–4 cases continued to show values just outside the RI.

**Fig. 3:**
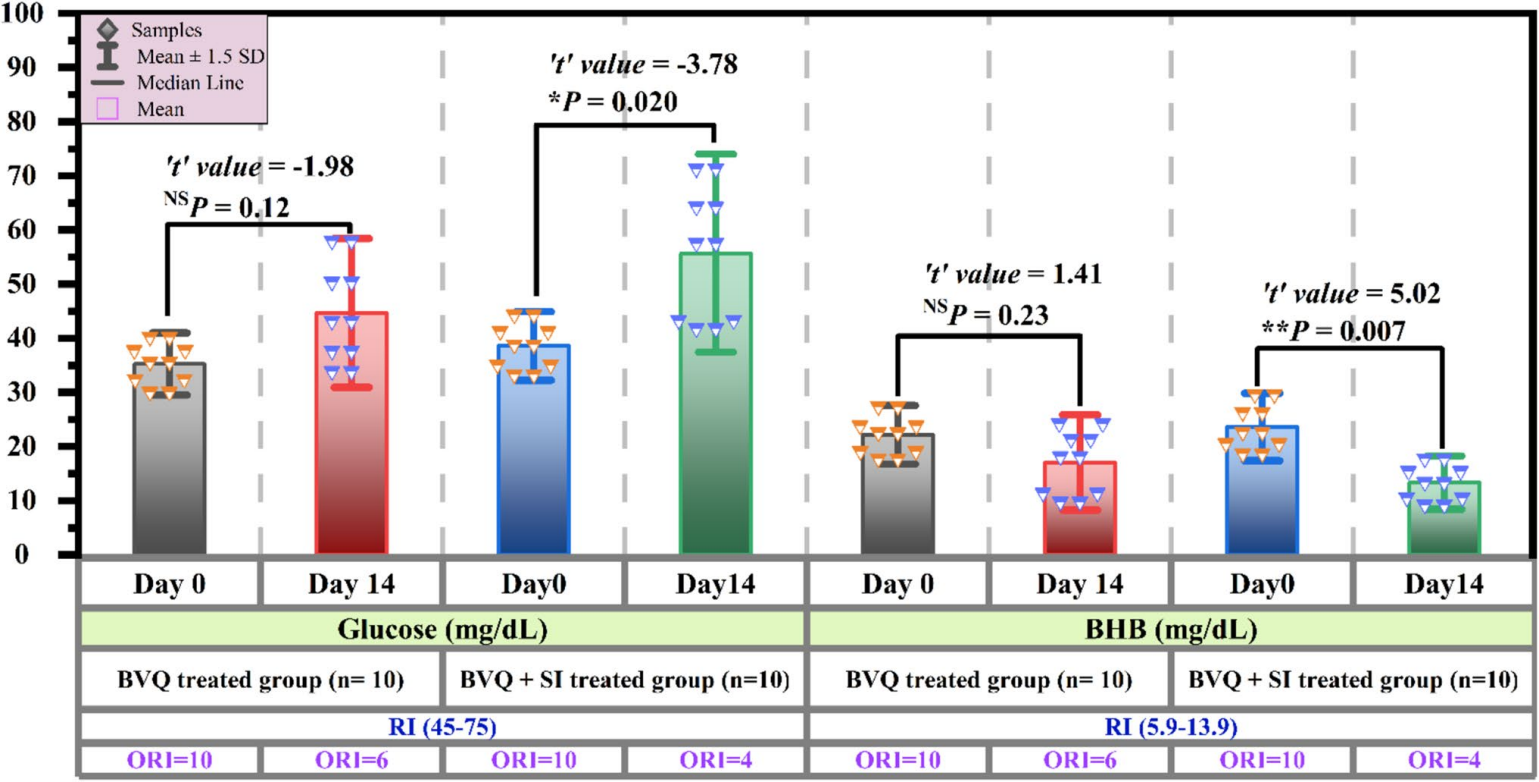
Bar plots depict the Mean ± SD of serum glucose and ketogenesis markers in cattle naturally infected with *T. annulata* before and after a two-week treatment with 2.5 mg/kg BVQ or a combination of 2.5 mg/kg BVQ and 2.23 mg/kg SI. Diamonds denote the individual value for each case in each group. Error bars represent ± standard deviation of the Mean, whereas the bar length indicates the mean value of each biomarker for each group. Paired Student’s *t*-test was used for comparisons of means in pretreatment Day 0 and posttreatment Day 14 within the same treatment group, with the significance levels versus pretreatment Day 0 as follows: ^NS^ *P* > 0.05; * *P* < 0.05; ** *P* ≤ 0.01. BHB: β-hydroxybutyrate

Illustrated bar plots in Figs. 2 and 3 showed the varied impacts of the two treatment regimens on reestablishing negative energy balance and ameliorating the metabolic switch towards ketogenesis observed on Day 0 in *T. annulata*-infected cows. Administration of 2.5 mg/kg BVQ did not yield significant differences (*P* > 0.05) in total cholesterol and glucose levels on Day 14 post-treatment, which remained below the RI. Similarly, BHB levels remained consistently elevated beyond the RI, with six cows continuing to exceed the RI. Indeed, the combination of 2.5 mg/kg BVQ and 2.23 mg/kg SI demonstrated superior efficacy, significantly elevating cholesterol and glucose levels (*P* < 0.05) while dramatically reducing BHB (*P* ≤ 0.01), normalizing these parameters in most of the studied cases. Despite this, four clinical cases continued to exhibit values ORI, suggesting that the combination therapy was more effective, but not entirely curative. Notably, triglyceride levels remained stable within the RI and did not change significantly (*P* > 0.05) between Day 0 and Day 14 in any treatment regimen.

Table 4 illustrates how BVQ-based therapy corrects protein metabolism and ameliorates inflammation in *T. annulata*-infected cows. Proteinogram revealed the prevalence of hypoalbuminemia, hyperglobulinemia, and decreased A/G ratios to modulate inflammatory events and combat the infection. Changes included increased levels of positive APPs, such as HP and α1-AGP, and reduced activity of negative APPs, like PON 1and albumin. On Day 14 post-treatment with 2.5 mg/kg BVQ, the existing inflammatory condition and dysproteinemia status did not show any significant amelioration when compared to the pre-treatment group. The concentration of negative acute phase reactants remained below the RI (*P* > 0.05). On Day 14 post-treatment, T. Protein and globulin levels exhibited significant reductions (*P* ≤ 0.01˗ ≤ 0.001); however, only globulin levels reverted to the RI, whilst T. Protein levels dropped below the RI. The A/G ratio was significantly elevated (*P* ≤ 0.001), attributable to compromised albumin production, which acts as a negative acute phase reactant. Notwithstanding a significant (*P* < 0.05) reduction in HP and α1-AGP levels, these values remained unchanged over the RI, indicating an unresolved inflammatory status in the BVQ-treated group.

**Table 4:** Serum proteinogram in naturally infected cattle with *T. annulata* before and after a two-week treatment with 2.5 mg/kg BVQ or a combination of 2.5 mg/kg BVQ and 2.23 mg/kg SI

Parameters	Reference Interval	BVQ treated group (*n* = 10)					BVQ + SI treated group (*n* = 10)				
		Day 0		Day 14		*‘t*’ value *(P*-value)	Day 0		Day 14		*‘t’* value (*P*-value)
		ORI	Mean ± SD	ORI	Mean ± SD		ORI	Mean ± SD	ORI	Mean ± SD	
T. Protein (g/dL)	5.7–8.10	0	7.19 ± 0.48	4	5.26 ± 0.92(**‣**)	t = 4.80 *P* = 0.01^**^	2	7.22 ± 0.98	2	6.33 ± 0.78	t = 2.14 *P* = 0.10^NS^
Albumin (g/dL)	2.1–3.6	8	1.67 ± 0.31(**‣**)	6	2.04 ± 0.28(**‣**)	t = −1.87 *P* = 0.14^NS^	6	1.93 ± 0.28(**‣**)	2	2.49 ± 0.30	t = −6.72 *P* = 0.003^**^
Globulin (g/dL)	2.8–5.4	8	5.53 ± 0.67(**‣**)	4	3.22 ± 0.75	t = 8.12 *P* = 0.001^***^	6	5.28 ± 0.81	0	3.85 ± 0.77	t = 3.07 *P* = 0.037^*^
A/G (ratio)	0.8–1.60	10	0.31 ± 0.11	9	0.65 ± 0.13	t = −11.27 *P* = 0.001^***^	10	0.37 ± 0.047(**‣**)	8	0.66 ± 0.14(**‣**)	t = −4.19 *P* = 0.012^**^
HP (µg/mL)	0.10–0.80	10	2.08 ± 0.61	6	1.09 ± 0.36	t = 3.41 *P* = 0.027^*^	10	2.23 ± 1.11(**‣**)	3	0.80 ± 0.48	t = 4.77 *P* = 0.009^**^
α−1 AGP (mg/mL)	0.16–0.72	10	1.77 ± 0.59	6	0.81 ± 0.28	t = 3.63 *P* = 0.022^*^	10	1.85 ± 0.67(**‣**)	4	0.60 ± 0.26	t = 4.53 *P* = 0.011^**^
PON1 (U/mL)	25–165	10	16.13 ± 5.21(**‣**)	6	24.33 ± 6.89(**‣**)	t = −3.06 *P* = 0.038^*^	10	16.92 ± 3.94(**‣**)	4	104.94 ± 50.65	t = −4.093 *P* = 0.011^**^

Paired Student’s *t*-test was used for comparisons of means in pretreatment day 0 and posttreatment day 14 within the same treatment group. Significance versus pretreatment day 0: * *p* < 0.05; ** *p* ≤ 0.01; *** *p* ≤ 0.001. Values out of reference interval are marked as (**‣**) symbol. *ORI* number of cows that had values out of the reference interval, *HP* haptoglobin, *α−1 AGP* alpha 1-acid glycoprotein, *PON1* paraoxonase 1

Restoring metabolic and inflammatory abnormalities seen on Day 0 was most effectively achieved with a combination of BVQ and SI. There was a significant increase in albumin levels (*P* ≤ 0.01), a decrease in globulin levels (*P* < 0.05), and a normalization of T. Protein levels to within the RI. Restored balance in protein metabolism and albumin production was reflected by the markedly improved A/G ratio (*P* ≤ 0.01). The levels of positive APPs, such as HP and α1-AGP, decreased significantly (*P* ≤ 0.01), returning to the normal range in most cases. On the other hand, PON-1 activity increased significantly (*P* ≤ 0.01), suggesting an improvement in the liver's ability to neutralize free radicals. Although the combination treatment regimen was more effective, the existence of 4–2 cows with ORI levels verified that it was not completely curative in resolving inflammation and restoring protein homeostasis within two weeks.

### Molecular analysis results

**Fig. 4:**
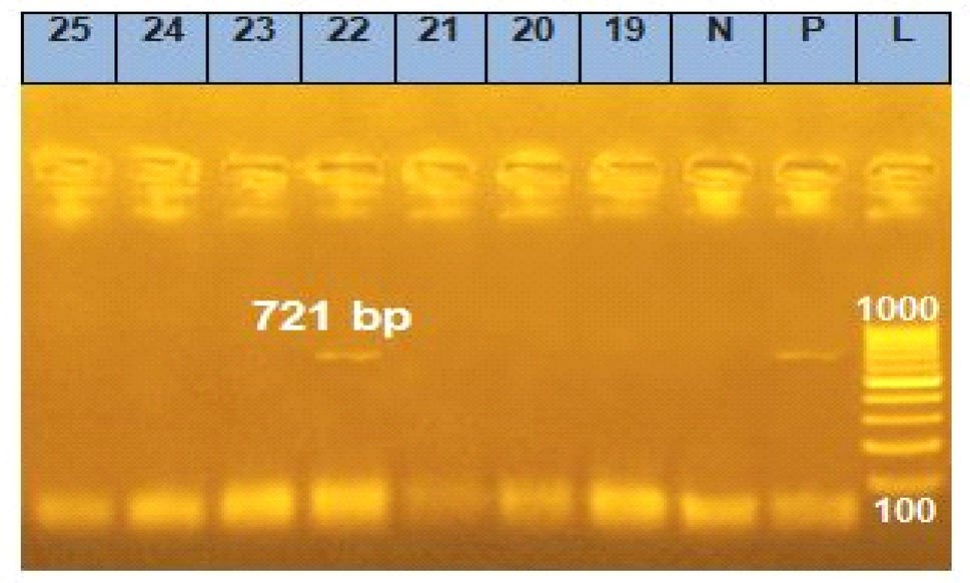
Amplification of the *T. annulata* Tams1 gene showing amplified target of 721 bp. M – 100 bp size DNA marker (Himedia, 100 lg/ml); Lane with sample number 22-positive sample

**Fig. 5:**
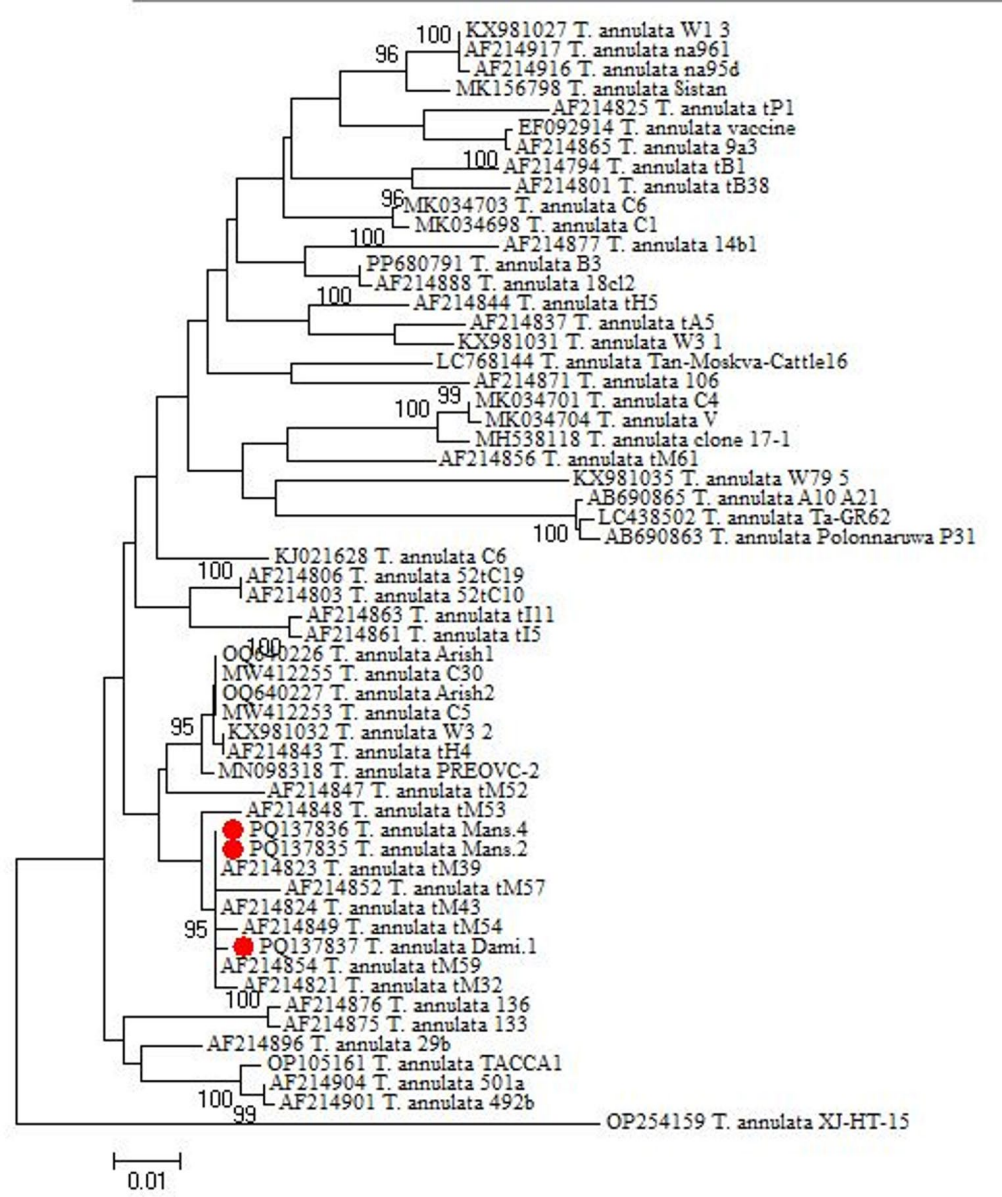
Phylogenetic analysis of the partial nucleotide sequences (721 bp) of the Tams 1 gene and other variants of *T. annulata* available in GenBank. Sequence isolated in Dakahlia and Damietta is indicated with the red circles

**Fig. 6:**
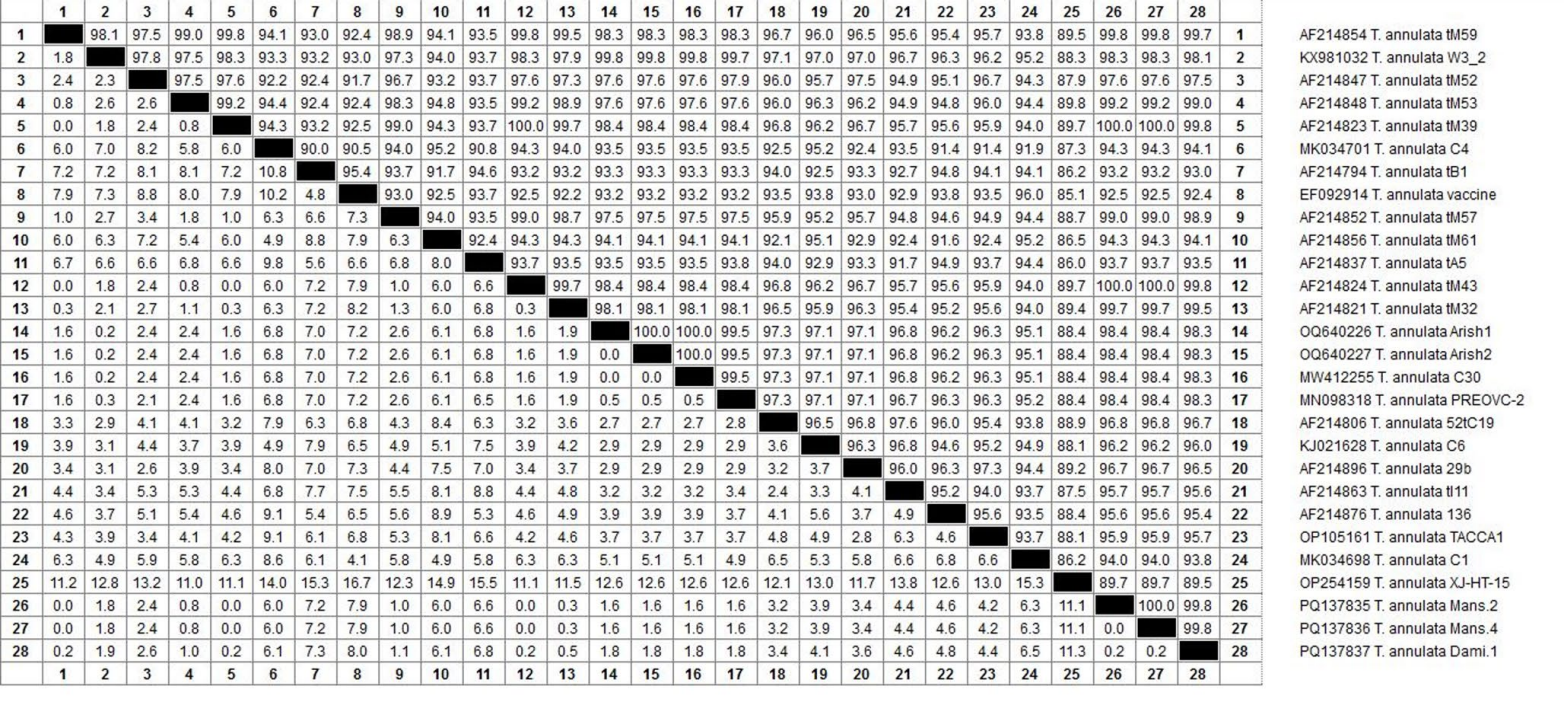
Tams 1 nucleotide difference estimation (lower left triangle) and percentage identity (upper right triangle) for *T. annulata* isolate from Dakahlia and Damietta Governorates, Egypt

PCR analysis of blood samples collected from Dakahlia and Damietta governorates detected positive theileriosis in 49 (32.9%). Amplification of *T. annulata* DNA revealed a product size of 721 bp, considered positive for *T. annulata* (Fig. 4). A higher genetic divergence was observed between Dakahlia and Damietta isolate (GenBank accession numbers PQ137836 and PQ137837) (Fig. 5). The amplicon size of the partial Tams 1 gene of *T. annulata* revealed 721 bp. Analysis of the Tams 1 gene partial nucleotide sequence of *T. annulata* of Damietta (PQ137837) through online BLAST showed a similarity of 99.7%, 98.3%, 98.3%, and 95.7% with Mauritania (AF214854), Arish, Egypt (OQ640227), Pakistan (MW412255) and Algeria (OP105161), respectively (Fig. 6). Analysis of the Tams 1 gene partial nucleotide sequence of *T. annulata* of Dakahlia (PQ137836) through online BLAST showed a similarity of 100%, 98.4%, 98.4%, and 96.2% with Mauritania (AF214824), India (MN098318), Arish, Egypt (OQ640227) and New Valley, Egypt (KJ021628), respectively (Fig. 6).

## Discussion

Tropical theileriosis leads to significant economic losses due to high mortality, decreased milk supply, slower growth rates, and higher treatment costs (Brown 1997)**.** Bovine theileriosis is a life-threatening disease that affects several animal species worldwide. In Egypt, this is a significant concern as it significantly impacts animal output and reproduction (Mohamed and Ebied 2014)**.** Updating the epidemiological status and identifying associated risk factors are critical for understanding disease dynamics and implementing effective control strategies. Hematological, biochemical, and molecular techniques were used to explore two governorates and the genetic diversity of the identified *T. annulata* genotypes.

The detected clinical signs of bovine theileriosis in our study were high fever and tick infestation, as well as lymph node enlargement, lacrimation, corneal opacity, and loss of appetite. Similar clinical manifestations were observed among Egyptian cow and buffalo populations (Al-Hosary et al. 2018; El-Dakhly et al. 2018; Yousef et al. 2020)**.** In this study, the prevalence of bovine theileriosis was higher in middle-aged cattle (1–3 years old; 61.9%) than in older cattle (3–8 years old; 34.6%) and younger cattle (less than 1 year old; 10.8%). This was comparable to the prior investigations (Selim et al. 2021; Hassan et al. 2024). The higher frequency in older animals may be due to increasing infection or management-related factors. Lower prevalence was seen in animals under one year, which is consistent with the findings of Kala et al. (2018).

The prevalence rate was higher in females (53.1%) than in males (46.9%), as shown in previous studies (Selim et al. 2022; Hassan et al. 2024). Many reports indicated that the high prevalence of bovine theileriosis in females is related to more hormonal fluctuations, weak immune response, and could be related to stress factors such as pregnancy, parturition, and milk production (Inci et al. 2008; Saeed et al. 2016; Mohammed-Ahmed et al. 2018; Tayebwa et al. 2018; Kebzai et al. 2024). Pregnant animals had a higher prevalence (69.4%) than non-pregnant animals (30.6%), and this was consistent with Selim et al. (2021). The current study found that the prevalence of bovine theileriosis was higher in tick-infested cattle (89.8%) than in non-infested cattle (10.2%), confirming the role of ticks in the dissemination of bovine theileriosis between animals (Al-Hosary et al. 2021; Abdel-Shafy et al. 2022)**.**

This study found that no ectoparasiticide treatment was consistently associated with greater *T. annulata* infection rates. Comparable findings were reported (Adjou Moumouni et al. 2018; Miyama et al. 2020). It also explains the significance of routine ectoparasiticide treatment, which is one of the main ways for treating the disease in cow herds (Jenkins 2018)**.** The Mediterranean climate of the present study is characterized by rainfall in the winter and high temperatures in summer. The seasonal findings of the present study demonstrated a markedly higher prevalence rate during summer (83.7%) compared to winter (16.3%), suggesting that the occurrence of infections is substantially greater in the warmer season. These observations agree with previous reports by Mohammad Al-Saeed et al. (2010), Abaker et al. (2017), Selim et al. (2020), and Zeb et al. (2020).

The hematological parameters of cattle affected RBC, Hb, and PCV values were significantly lower in cattle clinically afflicted with tropical theileriosis than in healthy animals, similar to (Abd Ellah 2015)**.** Conversely, as the parasite load grew, MCV values rose as well (Nazifi et al. 2010)**.** When compared to healthy animals, *T. annulata*-infected cattle also had neutropenia and eosinophilia (Ganguly et al. 2015)**.** The mean values in this study were within the normal range, which was consistent with the findings of another study conducted in Spain, even though several instances had low RBC values, as (García-Sanmartín et al. 2006)**.** These findings will aid the establishment of immediate and efficient control measures against tropical theileriosis. Pretreatment levels of biochemical values before treatment, such as ALT, AST, T. Bilirubin, and biliary (ALP, GGT) biomarkers in cows infected with *T. annulata,* were increased were related to Kachhawa et al. (2016). Decreased total cholesterol and glucose serum concentration may be due to the abnormalities in liver functions and/or abnormalities in metabolism, and anorexic state of affected animals impacts the two treatment regimens on reestablishing negative energy balance and ameliorating the metabolic switch towards ketogenesis observed on Day 0 in *T. annulata* infected cows near to (Tennant 1997; Sandhu et al. 1998; Radostits et al. 2000; Hussein et al. 2007; El-Deeb and Younis 2009; Kachhawa et al. 2016)**.** Proteinogram declared the prevalence of hypoalbuminemia, hyperglobulinemia, and decreased A/G ratios, possibly due to impaired hepatic metabolic function, and increased immunoglobulin production to modulate inflammatory events and fight the infection as (Yurtseven and Uysal 2009; Kachhawa et al. 2016; Prajapati et al. 2022).

Given that Dakahlia and Damietta Governorates, Egypt, are regarded as an endemic zone for tropical theileriosis, the high prevalence of *T. annulata* infection found by whole blood PCR analysis is not surprising. Our research will be useful in determining the frequency of *T. annulata* infections in Dakahlia and Damietta and in formulating a precise control strategy that will lessen the disease's financial costs. The prevalence of *T. annulata* in this study using the Tams 1 gene PCR assay in cattle and buffalo was 32.88% where the infection rate was 33.3% in Dakahlia and 32.2% in Damietta, and this was higher than that reported in Egypt, as in North Egypt, 11.6% (Rizk et al. 2017)**,** Alexandria 22.5% and Menofia 16.2% (Selim et al. 2022)**.** Our rate was lower than the rate of 63.6% reported in the El-Wady El-Gaded governorate in South Egypt (Al-Hosary et al. 2018)**.** and higher rates were reported (54.86%) in Odisha (India) (Selim et al. 2021)**.** Phylogenetic analysis of *T. annulata* isolated from Dakahlia and Damietta Governorates was successfully applied and showed relationships with other local and global isolates. The Tams 1 gene, a genetic marker, was amplified successfully, and the molecular phylogeny of *T. annulata* was created. The present isolate had close kinship with *T. annulata* from the Damietta, Egypt isolate (PQ137837) in this study, Mauritania (AF214854), Arish, Egypt (OQ640227), Pakistan (MW412255), and Algeria (OP105161). The Tams 1 gene, a genetic marker, was amplified successfully, and the molecular phylogeny of *T. annulata* was created. The present isolate had close kinship with *T. annulata* from Dakahlia, Egypt isolate (PQ137836) in this study, Mauritania (AF214824), India (MN098318), Arish, Egypt (OQ640227) and New Valley, Egypt (KJ021628). Our results were consistent with the molecular phylogeny of *T. annulata* based on the Tams 1 gene marker from Mauritania, Pakistan, and India that was published (Gubbels et al. 2000; Aparna et al. 2013; Sivakumar et al. 2014)**.**

In the present study, serum biochemical profile of the Buparvaquone group revealed that hepatic (ALT, AST, T. Bilirubin) and biliary (ALP, GGT) biomarkers in cows infected with *T. annulata* showed no significant improvement from pretreatment, were related to Piccione et al. (2010), Neamat-allah (2016), Panousis et al. (2018), and Güney and Şentürk (2023)**.** Compared to the combination therapy of BVQ + SI group demonstrated enhanced efficacy in hepatic and biliary health compromised by *T. annulata* infection, as indicated by the significant reduction in these parameters. The combination therapy of BVQ and SI demonstrated enhanced efficacy in hepatic and biliary health compromised by *T. annulata* infection, as indicated by the significant reduction in these parameters, normalizing them to within the RI for most cases. Effectiveness of SI treatment to prevent vascular formation in cancerous tissue. Toxicology studies in cattle have shown that SI has no toxic effects and is considered a safe drug in the treatment of liver disorders. The medicinal herb Silybum marianum's pure seed extract yields a flavonoid compound known as SI. Silymarin exhibited dose-dependent antioxidant and anti-apoptotic effects by modulating the functions of liver cell microsomes and nuclei, consistent with the findings of previous studies (Lee et al. 2004; Jayaraman and Namasivayam 2011; Borah et al. 2018; Khazaei et al. 2022).

## Conclusion

This study provides comprehensive and novel insights into bovine tropical theileriosis by integrating epidemiological risk factors, clinical manifestations, hematological and biochemical alterations, molecular characterization, and therapeutic efficacy. Seasonality, ocular cataracts, pregnancy status, tick infestation, and the frequency of ectoparasiticide application were identified as significant risk factors influencing disease occurrence. Infected animals exhibited marked hematological and biochemical disturbances, reflecting the systemic nature of the disease, particularly its adverse effects on immune competence and hepatic function. Notably, the combination therapy of buparvaquone (BVQ) and supportive immuno-hepatic intervention (SI) proved superior to monotherapy, offering enhanced parasitic clearance alongside improved liver recovery and immune regulation. These findings highlight the importance of adopting integrated control strategies that combine effective tick management, optimized husbandry practices, molecular diagnostic tools, and evidence-based combination treatments. Implementation of such approaches will be pivotal in reducing disease prevalence, mitigating economic losses, and improving animal health, welfare, and productivity in Egypt and other Theileria-endemic regions.

## Data Availability

Data is available within the article to support the findings of this study.
